# Acute and Subacute Oral Toxicity Assessment of Kinkeliba (*Combretum micranthum* G. Don) Ethanolic Extract in BALB/c Mice

**DOI:** 10.3390/plants14121776

**Published:** 2025-06-10

**Authors:** Ibrahima Mamadou Sall, Alina Diana Haşaş, Amiali Malek, Dan Cristian Vodnar, Meriem Aziez, Ecaterina Semzenisi, Dragoş Hodor, Romelia Pop, Alexandru-Flaviu Tăbăran

**Affiliations:** 1 Department of Anatomic Pathology, Faculty of Veterinary Medicine, University of Agricultural Sciences and Veterinary Medicine of Cluj-Napoca, 400372 Cluj-Napoca, Romania; ecaterina.semzenisi@student.usamvcluj.ro (E.S.);; 2 Department of Pathophysiology, Faculty of Veterinary Medicine, University of Agricultural Sciences and Veterinary Medicine of Cluj-Napoca, 400372 Cluj-Napoca, Romania; 3 Laboratory of Food Technology and Human Nutrition, National Higher School of Agronomy (ENSA), Algiers 1603, Algeria; 4 Department of Food Science, Faculty of Food Science and Technology, University of Agricultural Sciences and Veterinary Medicine Cluj-Napoca, 400372 Cluj-Napoca, Romania; dan.vodnar@usamvcluj.ro; 5 Laboratory of Plant Biotechnology and Ethnobotany, Faculty of Nature and Life Sciences, University of Bejaia, Bejaia 06000, Algeria; meriem.aziez@univ-bejaia.dz

**Keywords:** *Combretum micranthum*, kinkéliba, acute toxicity, subacute toxicity, ethanolic extract, BALB/c mice

## Abstract

*Combretum micranthum* G. Don (kinkeliba) is a medicinal plant traditionally employed in West Africa for its diuretic and gastrointestinal therapeutic properties. Despite its extensive ethnomedicinal use, comprehensive toxicological assessments are still lacking. This study aimed to characterize the phenolic composition of *C. micranthum* ethanolic leaf extract using HPLC-DAD-ESI-MS and evaluate its acute and subacute oral toxicity in BALB/c mice, per OECD Guideline 420. Female mice received oral doses of 50, 300, and 2000 mg/kg of extract for acute toxicity assessment for 14 days. In the subacute study, both sexes were administered daily doses at the same concentrations over 28 days. Clinical signs, body weight, and food and water consumption were regularly monitored throughout both protocols. At the end of each study, hematological, biochemical, and histopathological parameters were analyzed. Phenolic profiling revealed nine major compounds with a total of 293.54 mg/g extract. No mortality or significant clinical manifestations were observed at any dose. However, significant variations in platelet counts and amylase activity were noted in the acute phase. In the subacute model, slight, non-critical alterations in hepatic and renal biomarkers were observed, without signs of systemic toxicity. Histopathological examination revealed similar lesions in both acute and subacute phases, including multifocal inflammatory infiltrates (lymphocytes and neutrophils) in the periportal area of the liver, minimal bacterial overgrowth in the superficial layer of the gastric mucosa, minimal medullary mineralization and inflammatory infiltrates with lymphocytes in the kidneys, and minimal to moderate vacuolization in the pancreatic acini. These results indicate that *C. micranthum* ethanolic extract is relatively safe at the tested doses, reinforcing its traditional use and supporting further research into its pharmacological potential.

## 1. Introduction

Medicinal plants are extensively used worldwide as traditional remedies, playing a vital role in human and animal healthcare systems. They are valued not only for treating various ailments but also for maintaining overall health [1]. Their significance lies in their integration into natural, cultural, and traditional practices, thus representing a promising avenue for the discovery of novel therapeutic agents. According to the World Health Organization, over 80% of the global population relies on traditional medicine for primary healthcare needs, with a particularly strong reliance observed in African communities where traditional medicine is deeply embedded in cultural heritage [2].

The *Combretaceae* family comprises around 600 species of trees, shrubs, and lianas, distributed into about 20 genera, primarily thriving in tropical and subtropical regions of Africa and India [3]. The *Combretum* genus includes approximately 250 species, which are widely distributed in these regions [4].

*Combretum micranthum*, known as kinkéliba, is a medicinal plant native to Northwest Africa [5]. Its use in traditional medicine dates back centuries and has been recognized in the French pharmacopeia since 1937 and the African pharmacopeia since 1985 [6]. This wild shrub, which grows to heights of approximately 4 to 5 m, is adapted to arid climates and is commonly found in Mauritania, Senegal, Sierra Leone, Guinea, Mali, Gambia, Niger, Côte d’Ivoire, Ghana, Nigeria, and Benin [6].

In traditional medicine, the leaves of *Combretum micranthum* are widely used in herbal drinks, teas, and infusions to manage various health issues, including cough, bronchitis, malaria, high blood pressure, fever, diarrhea, anemia, and gastrointestinal disorders such as colic and vomiting [7,8]. Recent research has identified over 150 organic compounds in its extracts, including 34 flavonoids, 16 phenolic acids, 14 alkaloids, 15 fatty acids, 14 terpenoids/steroids, 24 amino acids, 8 carbohydrates, and 6 minerals (e.g., calcium, magnesium, potassium, sodium, and iron), and additional 30 organic compounds have been identified from this plant [1]. These newly identified compounds in *Combretum micranthum* leaf extracts have been shown to exhibit antibacterial [9,10], antiviral, anti-inflammatory [11], cholagogue [12], hepatoprotective [13], moderately antimalarial [6,9], diuretic [14], and antidiabetic properties [15].

Despite these benefits, medicinal plants present potential risks due to their bioactive compounds, which, although therapeutic, can be toxic under certain circumstances. Scientific evaluation of traditional plants, such as *Combretum micranthum*, is essential to ensure their safe use within communities [16]. In-depth toxicological studies are necessary to assess safety profiles, establish safe doses, and better understand their mechanisms of action [17]. The originality of this study lies in the in-depth analysis of biological and histopathological parameters, an aspect often overlooked in previous research on the toxicity of *Combretum micranthum* [10,18,19]. These findings open new avenues and provide valuable insights for future research into the toxicity of this plant.

Thus, this study focuses on the comprehensive phenolic profiling of the ethanolic extract of *Combretum micranthum* using High-Performance Liquid Chromatography with Diode Array Detection coupled with Electrospray Ionization Mass Spectrometry (HPLC-DAD-ESI/MS) and investigates its acute (14-day) and subacute (28-day) oral toxicity in BALB/c mice. Toxicity is evaluated through clinical, biochemical, hematological, and histological assessments, addressing both local and systemic effects. This dual approach aims to establish a robust safety profile, identify potential adverse effects, and provide critical data to support the plant’s therapeutic potential.

## 2. Results

### 2.1. Extraction Yield

The yield of the ethanolic extract of *Combretum micranthum* is presented in Table 1.

### 2.2. Phenolic Composition of the Ethanolic Extract of *Combretum micranthum*

The phenolic composition of the ethanolic extract of *Combretum micranthum* (Table 2) was determined, revealing the presence of nine major compounds, with a total phenolic content of 293.54 mg/g of extract. The most abundant compound identified was sanguiin H-4, combretastatin B1, and corilagin. Significant amounts of ellagic acid and its glycosylated derivatives, ellagic acid-arabinoside and ellagic acid-glucoside, were also detected. Additionally, lower concentrations of protocatechuic acid, 1,6-digalloyl-glucose, and gallic acid were identified. This phenolic profile highlights the predominance of ellagitannins and stilbenes, as well as hydroxybenzoic acids and gallotannins, in the extract.

### 2.3. Acute Toxicity

#### 2.3.1. Zootechnical Parameters

**Figure 1:**
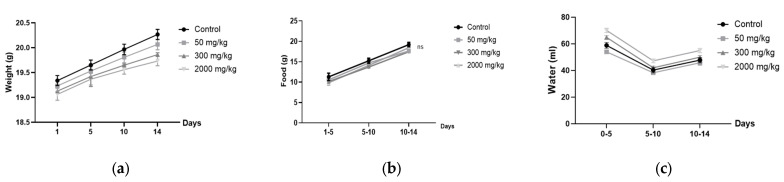
Zootechnical parameters in female BALB/c mice: (**a**) weight gain; (**b**) food consumption; (**c**) water intake.

The results, shown in Figure 1, indicate that the zootechnical parameters, including body weight gain, food intake, and water consumption, were monitored in female (Figure 1a–c) BALB/c mice. No significant differences (*p* > 0.05) were observed between the different treatment groups (50, 300, 2000 mg/kg) and the control group over the 14-day period.

#### 2.3.2. Organ Weight

The results of the statistical analysis of organ weights (lungs, liver, heart, and spleen) show no significant difference (*p* > 0.05) between the test and control groups, with means ± standard deviations presented in Table 3.

#### 2.3.3. Biochemical Parameters

The biochemical analysis conducted in the acute toxicity study (Table 4) revealed that the administration of ethanolic extract at doses of 50, 300, and 2000 mg/kg did not induce any statistically significant alterations (*p* > 0.05) in most serum biochemical parameters when compared to the control group. These parameters included albumin (ALB), total proteins (TP), globulins (GLOB), albumin/globulin ratio (A/G), alanine aminotransferase (ALT), alkaline phosphatase (ALP), total bilirubin (TB), urea (UREA), creatinine (CREA), glucose (GLU), phosphate (PHOS), potassium (K^+^), sodium (Na^+^), and serum calcium (Ca), indicating preserved hepatic, renal, and metabolic functions. However, statistically a significant increase (*p* < 0.001) in serum amylase (AMY) levels was observed in all treated groups (50 mg/kg: 2734.66 ± 10.57 u/L; 300 mg/kg: 2731.66 ± 12.57 u/L; 2000 mg/kg: 2739.66 ± 18.57 u/L) compared to the control group (2714.66 ± 11.15 u/L), suggesting a possible stimulatory effect of the extract on pancreatic enzymatic activity. Despite this elevation, the absence of other clinical or biochemical signs of pancreatic damage supports the hypothesis of a non-toxic adaptive physiological response.

#### 2.3.4. Hematological Parameters

The hematological analysis in the acute toxicity study (Table 5) showed no significant differences (*p* > 0.05) between the treated groups (50 mg/kg, 300 mg/kg, and 2000 mg/kg) and the control group for most parameters, including white blood cells (WBC), lymphocytes (LYM), monocytes (MON), neutrophils (NEU), red blood cells (RBC), hemoglobin (HGB), and hematocrit (HCT). However, statistically a significant decrease in platelet count PLT (*p* < 0.001) was observed in the treated groups, with values of 372 ± 4.35 × 10⁹/L, 335.66 ± 2.5 × 10⁹/L, and 380.66 ± 1.55 × 10⁹/L for 50, 300 and 2000 mg/kg, respectively, compared to the control group (574 ± 3.60 × 10⁹/L). This suggests that the ethanolic extract may affect platelet levels, although no other hematological changes were observed.

#### 2.3.5. Histopathological Analysis

**Figure 2:**
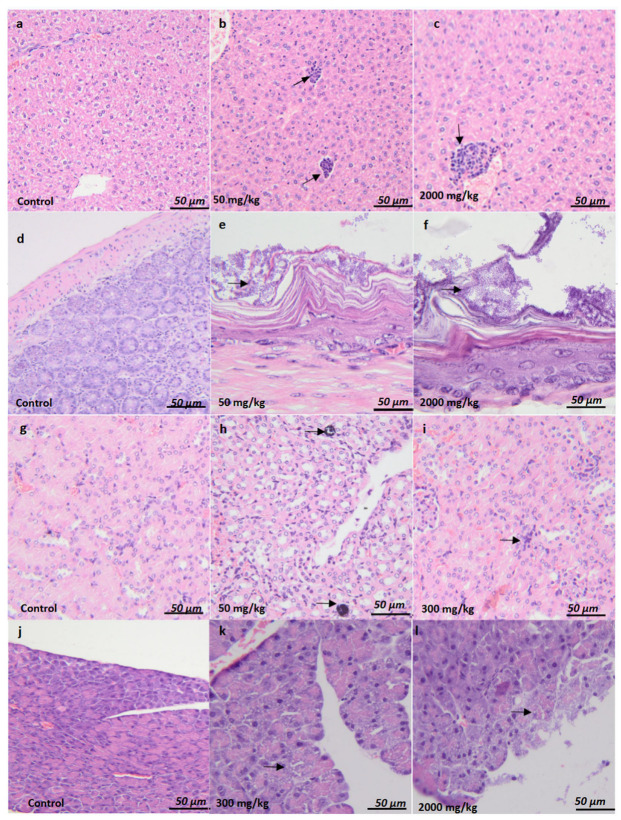
Histological evaluation of organ-specific acute toxicity and sub-chronic toxicity in BALB/c female and male mice. Liver (**a**–**c**). No significant findings (**a**). Multifocal mixed (lymphocytes and neutrophils) inflammatory infiltrate was observed in the periportal area (arrow) (**b**,**c**). Stomach (**d**–**f**). No significant findings (**d**). Minimal bacterial overgrowth was observed in the superficial layer of the gastric mucosa (arrow) (**e**,**f**). Kidney (**g**–**i**). No significant findings (**g**). Minimal medullary mineralization (arrow) (**h**), an inflammatory infiltrate with lymphocyte cells was observed (arrow) (**i**). Pancreas (**j**–**l**). No significant findings (**j**). Minimal to moderate vacuolization in the pancreatic acini (arrow) (**k**,**l**). H&E stain. Ob.x 10 (**a**,**g**), ob.x 20 (**b**), ob.x 40 (**c**,**d**,**h**–**j**), ob.x 100 (**e**,**f**,**k**,**l**), Scalebar: 80 µm (**a**,**g**), 60 µm (**b**). 40 µm (**c**,**d**,**h**–**j**), 10 µm (**e**,**f**,**k**,**l**).

The histopathological evaluation of acute toxicity revealed minimal to moderate changes in multiple organs (Figure 2). In the liver, periportal regions exhibited multifocal mixed inflammatory infiltrates consisting of lymphocytes and neutrophils, while the remaining parenchyma showed no significant findings. The gastric mucosa, within the nonglandular segment, showed minimal bacterial overgrowth in the superficial layer, with no other significant alterations. In the kidney, minimal medullary mineralization and a mild inflammatory infiltrate with lymphocytes were noted. The pancreas demonstrated minimal to moderate vacuolization within the acini, whereas the remaining pancreatic parenchyma appeared histologically normal. Corresponding semi-quantitative scores for these findings are summarized in Table 6.

### 2.4. Sub-Chronic Toxicity

#### 2.4.1. Zootechnical Parameters

**Figure 3:**
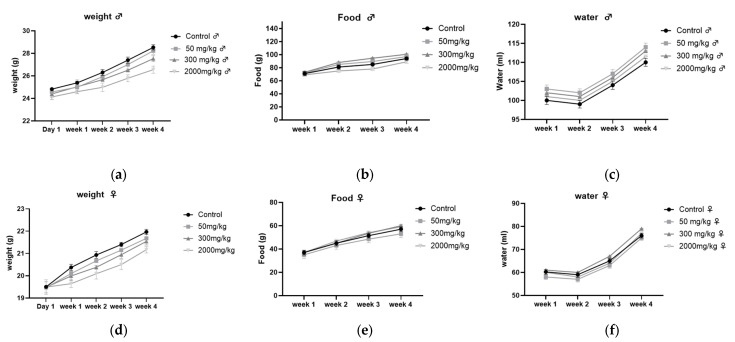
Zootechnical parameters in male (**a**–**c**) and female (**d**–**f**) BALB/c mice: (**a**,**d**) weight gain, (**b**,**e**) food consumption, (**c**,**f**) water intake.

The results, shown in Figure 3, indicate that the zootechnical parameters, including body weight gain, food intake, and water consumption, were monitored in male (Figure 3a–c) and female (Figure 3d–f) BALB/c mice. No significant differences (*p* > 0.05) were observed between the different treatment groups (50, 300, 2000 mg/kg) and the control group over the 28 days, with all parameters showing a progressive increase throughout the study.

#### 2.4.2. Organ Weight

The results of the organ weight analysis in male and female BALB/c mice showed no significant differences (*p* > 0.05) between the test and control groups. The corresponding means ± standard deviations are provided in Table 7 and Table 8.

#### 2.4.3. Hematological Analysis of Female Mice in BALB/c

The Hematological Analysis results of female mice revealed that the results for (WBC, LYM, MON, Neu, RBC, MO, NEU, HGB, MCV, MCH, MCHC, PCT, MPV and RDW) showed no significant difference (*p* > 0.05) between the different concentration groups (50 mg/kg, 300 mg/kg, 2000 mg/kg) and the control group, as indicated by their means ± standard deviations in Table 9.

However, statistically significant changes were observed in the following parameters:

LY% showed statistically a significant decrease (*p* > 0.001) in the treated groups, with the following mean values ± standard deviations: 50 mg/kg (50 ± 0.90%), 300 mg/kg (65.80 ± 0.64%), and 2000 mg/kg (69.70 ± 0.90%), compared to the control group (76.0 ± 0.20%).

HCT showed statistically a significant decrease (*p* > 0.001) in the treated groups: 50 mg/kg (36.80 ± 0.25%), 300 mg/kg (27.84 ± 0.23%), and 2000 mg/kg (30.65 ± 0.63%), compared to the control group (51.08 ± 0.86%).

PLT showed a statistically significant decrease (*p* > 0.001) in the treated groups: 50 mg/kg (533 ± 3.60 × 10^9^/L), 300 mg/kg (286 ± 4.20 × 10^9^/L), and 2000 mg/kg (220 ± 2.10 × 10^9^/L), compared to the control group (625 ± 3.83 × 10^9^/L).

#### 2.4.4. Hematological Analysis of Male Mice in BALB/c

Statistical analysis of male mice results showed similar trends to those observed in males, except for a few differences. No significant differences (*p* > 0.05) were found between the different concentration groups (50 mg/kg, 300 mg/kg, 2000 mg/kg) and the control group, with means ± standard deviations presented in Table 10. Hematological parameters such as (WBC, LYM, MON, Neu, Neu %, RBC, HGB, MCV, MCH), MCHC, and MPV) showed no significant changes.

However, significant changes were observed in the following parameters:

LY% percentage in the treated groups showed a statistically significant decrease (*p* > 0.001) compared to the control group. The mean ± standard deviations for the treated groups were: 50 mg/kg (39.2 ± 0.23%), 300 mg/kg (24.3 ± 0.30%), and 2000 mg/kg (37.2 ± 0.60%), compared to the control group (62.2 ± 0.50%).

HCT showed statistically a significant increase (*p* > 0.001) in the treated groups, with the following mean values ± standard deviations: 50 mg/kg (59.89 ± 0.51%), 300 mg/kg (60.38 ± 0.14%), and 2000 mg/kg (59.88 ± 0.25%), compared to the control group (51.51 ± 0.70%).

Platelets also showed statistically a significant increase (*p* > 0.001) in the treated groups, with means ± standard deviations: 50 mg/kg (584 ± 6.1 × 10^9^/L), 300 mg/kg (505 ± 2.14 × 10^9^/L), and 2000 mg/kg (543 ± 0.25 × 10^9^/L), compared to the control group (347 ± 4.3 × 10^9^/L).

#### 2.4.5. Biochemical Analysis in Female BALB/c Mice

The statistical analysis of biochemical results in female mice exposed to subacute toxicity revealed no significant differences (*p* > 0.05) for parameters such as (ALB, PT, GLOB, A/G), TB, CREA, PHOS, K^+^, Na^+^, and Ca) among the different concentration groups (50 mg/kg, 300 mg/kg, 2000 mg/kg) compared to the control group.

However, statistically significant changes were observed in the following parameters:

ALT levels showed statistically a significant increase (*p* > 0.05) in the treatment groups, with mean values and standard deviations as follows: 50 mg/kg (93.33 ± 0.18 u/L), 300 mg/kg (73.0 ± 0.66 u/L), and 2000 mg/kg (92.00 ± 1.15 u/L), compared to the control group (33.33 ± 0.40 u/L).

ALP levels also showed statistically a significant increase (*p* > 0.001) in the treated groups: 50 mg/kg (104 ± 0.61 u/L), 300 mg/kg (104.66 ± 0.89 u/L), and 2000 mg/kg (103.66 ± 0.57 u/L), compared to the control group (87.0 ± 0.67 u/L). AMY levels increased statistically significantly (*p* > 0.001) across the different concentrations, with mean values: 50 mg/kg (2350.0 ± 13.37 u/L), 300 mg/kg (2355.0 ± 11.57 u/L), and 2000 mg/kg (2336.0 ± 90 u/L), compared to the control group (2156.66 ± 13.25 u/L). UREA levels statistically significantly decreased (*p* > 0.001) in the treated groups, with mean values: 50 mg/kg (44.85 ± 0.03 mg/dL), 300 mg/kg (44.10 ± 0.60 mg/dL), and 2000 mg/kg (29.21 ± 0.02 mg/dL), compared to the control group (56.27 ± 0.56 mg/dL).GLU levels also showed statistically a significantly increase (*p* > 0.001) in the treatment groups, with mean values: 50 mg/kg (240.25 ± 0.01 mg/dL), 300 mg/kg (249.45 ± 0. mg/dL), and 2000 mg/kg (240.40 ± 0.05 mg/dL), compared to the control group (120.82 ± 0.03 mg/dL), as shown in Table 11.

#### 2.4.6. Biochemical Analysis in Male BALB/c Mice

The statistical analysis of biochemical results in male mice subjected to subacute toxicity, considering parameters such as (ALB, TP), GLOB, A/G, TB, CREA, PHOS, K^+^, Na^+^, and Ca) revealed no significant differences (*p* > 0.05) among the different concentration groups (50 mg/kg, 300 mg/kg, 2000 mg/kg) compared to the control group.

However, significant changes were observed in the following parameters:

ALT levels showed a significant increase (*p* > 0.001) in the treatment groups, with mean values and standard deviations as follows: 50 mg/kg (107 ± 0.36 u/L), 300 mg/kg (181 ± 0.57 u/L), and 2000 mg/kg (142 ± 0.66 u/L), compared to the control group (38 ± 0.36 u/L). 

ALP levels demonstrated a significant increase (*p* > 0.001) in the treated groups, with mean values: 50 mg/kg (108 ± 0.02 u/L), 300 mg/kg (100 ± 0.66 u/L), and 2000 mg/kg (109 ± 0.66 u/L), compared to the control group (88 ± 0.12 u/L).AMY levels also showed a significant increase (*p* > 0.001) across the different concentration groups, with mean values: 50 mg/kg (2461.17 ± 9.31 u/L), 300 mg/kg (2453 ± 11.32 u/L), and 2000 mg/kg (2422 ± 12.57 u/L), compared to the control group (2196 ± 10.11 u/L).

UREA levels exhibited a significant decrease (*p* > 0.001) in the treatment groups, with mean values: 50 mg/kg (50.58 ± 0.49 mg/dL), 300 mg/kg (50.20 ± 0.07 mg/dL), and 2000 mg/kg (50.18 ± 0.90 mg/dL), compared to the control group (57.71 ± 0.60 mg/dL). GLU levels showed statistically a significant increase (*p* > 0.001) in the different concentration groups, with mean values: 50 mg/kg (147.25 ± 0.03 mg/dL), 300 mg/kg (145.94 ± 0.04 mg/dL), and 2000 mg/kg (143.48 ± 0.70 mg/dL), compared to the control group (126.03 ± 0.02 mg/dL), as shown in Table 12.

#### 2.4.7. Histopathological Analysis

The histopathological findings associated with acute and chronic toxicity are summarized in Table 13 and illustrated in Figure 2. In female BALB/c mice, multifocal mixed inflammatory infiltrate was observed in the periportal area observed in the liver at 2000 mg/kg (Figure 2c) compared to controls (Figure 2a), along with Minimal bacterial overgrowth was observed in the superficial layer of gastric mucosa (arrow) at 50 mg/kg (Figure 2e) and 2000 mg/kg (Figure 2f), compared to controls (Figure 2d). In male mice, a similar multifocal mixed inflammatory infiltrate was observed in the periportal area were observed in the liver at 50 mg/kg (Figure 2b) and 2000 mg/kg (Figure 2c), the gastric mucosa, within the nonglandular segment, showed minimal bacterial overgrowth in the superficial layer, with no other significant alterations (arrow) compared to controls (Figure 2d) and minimal to moderate vacuolization in the pancreatic acini at 300 mg/kg (Figure 2k) compared to controls (Figure 2j).

## 3. Discussion

The use of medicinal plants raises concerns about their safety, as herbalists often prepare these remedies without formal scientific training. Moreover, the lack of standardized preparation methods and dosage further complicates their application in clinical practice. Therefore, in-depth scientific studies and the establishment of strict regulations are necessary to ensure the safety and efficacy of these treatments, alongside clear labeling and quality control of herbal products [20].

HPLC-DAD-ESI-MS analysis of the *Combretum micranthum* ethanolic leaf extract revealed a rich and structurally diverse phenolic profile, reaching 293.54 mg/g of extract. Precise characterization of these phenolic constituents is critical, as such bioactive molecules exert multifaceted roles in modulating redox homeostasis, regulating enzymatic pathways, and attenuating inflammatory signaling cascades. These mechanisms, in turn, significantly influence toxicodynamic responses and contribute to the overall systemic tolerability and safety profile of phytopharmaceutical formulations [21,22].

However, it is important to acknowledge the limitations inherent in the analytical approach employed in this study. While HPLC-DAD-ESI-MS provides valuable preliminary insights into the phenolic composition, this technique, particularly when coupled with single quadrupole mass spectrometry, does not afford definitive structural elucidation of isobaric compounds or closely related glycosides. For instance, compounds such as ellagic acid and quercetin share identical molecular masses and UV spectra, complicating their discrimination without tandem mass spectrometric (LC-MS/MS) fragmentation data.

Among the identified compounds, sanguiin H-4 (102.56 mg/g) was predominant. This high-molecular-weight ellagitannin undergoes extensive microbial metabolism in the gut, yielding urolithins—metabolites with recognized safety profiles and beneficial bioactivities [23,24]. Combretastatin B1 (68.71 mg/g), a microtubule-disrupting stilbene with known cytotoxic effects in vitro, is characterized by poor oral bioavailability and rapid metabolism, limiting its systemic toxicity when administered orally [25]. Corilagin (63.29 mg/g), another abundant ellagitannin, has shown a favorable safety profile in rodents and exhibits hepatoprotective and anti-inflammatory properties at low to moderate doses [26,27]. Other phenolic acids and flavonoids identified, such as ellagic acid, gallic acid, and protocatechuic acid, are known to exert strong antioxidant effects. Yet, their biological impact depends largely on dose, chemical form, and interactions with gut microbiota [28,29].

To strengthen the chemical identification and validate the presence of key phenolic constituents, future studies employing LC-MS/MS or high-resolution tandem mass spectrometry are warranted. Such advanced methodologies would enable unequivocal discrimination of structural isomers and glycosylated derivatives, thereby increasing confidence in compound annotation and supporting more precise correlations with biological activities and safety outcomes.

Furthermore, the phenolic composition of the extract in our study aligns closely with the results reported by Zannou et al. [30], who performed solvent-based profiling of *C. micranthum* leaves and identified gallic acid, catechin, caffeic acid, and quercetin-3-glucoside as key constituents in ethanolic extracts, using a comparable 1:20 extraction ratio. Such consistency across studies reinforces the reproducibility of the phenolic fingerprint of this species and enhances the reliability of toxicological assessments.

In this study, the oral administration of ethanolic extract from *Combretum micranthum* leaves at doses up to 2000 mg/kg did not result in any mortality, either over a short period (14 days) or a prolonged period (28 days) in BALB/c mice. Previous studies [10,18,19,20] have shown similar results. Furthermore, zootechnical parameters (food and water consumption or weight gain) did not show significant changes in acute and subacute toxicity in male and female mice. The study conducted by Kpemissi et al. (2020) [19], whose results are consistent with ours. Oral administration at a dose of up to 5000 mg/kg showed no significant changes in food and water consumption, nor any treatment-related mortality for acute toxicity. Furthermore, oral administration at doses of 500 and 1000 mg/kg showed no signs of toxicity or treatment-related mortality over 28 days for subacute toxicity [19]. This macroscopically explains that these doses do not cause the death of the mice, nor the appearance of clinical signs of toxicity.

Biological analysis, particularly of biochemical parameters, revealed an increase in amylase parameters for both acute and subchronic toxicity. This elevation, observed in both male and female mice after administration of *Combretum micranthum* extract, may indicate an adaptive response of the digestive system or mild pancreatic irritation. Therefore, this increase is more likely to be a normal metabolic response than indicative of a major pancreatic deficiency [31].

In both males and females, a significant rise in alanine aminotransferase levels was observed in mice that received the ethanolic extract of *Combretum micranthum* in the context of subchronic toxicity. This increase could be interpreted as an adaptive response of the liver, rather than indicating severe toxicity, or may suggest hepatic stress, possibly due to the enhanced stimulation of hepatic enzymes by bioactive compounds such as flavonoids. These compounds, including sanguiin H-4 and combretastatin B1, are known for their low toxicity and side effects, despite their high concentrations that may interact with liver cells [32,33]. It is also interesting to note that an increase in ALT levels was observed in females. Hormonal differences between sexes could influence these variations [34].

An increase in alkaline phosphatase levels was observed in both male and female mice that received the ethanolic extract of *Combretum micranthum*. This may be associated with increased metabolic activity in the liver or with hepatic stress or other organs expressing this enzyme, indicating an adaptive response or cellular regeneration [35]. Although the extract is generally considered safe, it may temporarily stimulate hepatic enzymes, particularly those influenced by flavonoids and polyphenols, without leading to significant hepatic toxicity [36].

The decrease in urea levels observed in mice receiving the ethanolic extract of *Combretum micranthum* could indicate several toxicity-related phenomena. This decrease could signal renal dysfunction, impairing the kidneys’ ability to eliminate urea or suggest a protein metabolism shift, leading to reduced urea production. Liver dysfunction may also play a role, as the liver synthesizes urea. Finally, this decrease could reflect an adaptive metabolic response to the extract, though further investigations are needed to confirm the exact cause [37].

The increase in glucose levels observed in mice that received the ethanolic extract of *Combretum micranthum* could indicate an immune or inflammatory response to the administration of this extract. This elevation may be due to immune system activation in response to bioactive compounds in the extract, such as flavonoids (quercetin, kaempferol, or luteolin) and other molecules (tannins, saponins, or alkaloids), which could be perceived as foreign agents by the body, triggering an increase in glucose production. This increase could also indicate mild inflammation or a defense mechanism against potential subclinical toxicity of the administered compound [38,39].

Regarding the hematological parameters, the decrease in lymphocyte production observed in both male and female mice treated with the ethanolic extract of *Combretum micranthum* for subchronic toxicity may indicate immune system activity suppression, which could be caused by the bioactive compounds present in the extract. Certain bioactive compounds may interfere with the production or maturation of immune cells in the bone marrow, leading to a reduction in lymphocytes, which in turn could increase the organism’s vulnerability and decrease immune system efficiency [40,41]. Some flavonoids, such as tilianin and vitexin, may be responsible for the suppression of immune system activity. Although these compounds have beneficial effects in reducing excessive inflammation, they can also inhibit lymphocyte proliferation at high concentrations or during prolonged exposure, disrupt cytokine production, or interfere with the maturation of immune cells in the bone marrow. Such excessive modulation could lead to a decrease in circulating lymphocytes, as observed in the study.

The increase in hematocrit levels in male mice and decrease in females after administration of ethanolic extract of *Combretum micranthum* for sub-chronic toxicity suggests a sex-differentiated physiological response. This variation could be attributed to hormonal effects, differences in red blood cell production regulation, or sex-specific physiological or toxic stress responses. Further studies are needed to better understand the mechanisms underlying these observations, and these may be considered about immune function and potential toxicity [42,43].

There was an increase in platelet levels in male mice and a decrease in females after administration of the ethanolic extract of *Combretum micranthum.* Platelet levels typically differ between sexes, with males generally showing higher values. This difference may be explained by hormonal factors, notably the stimulatory effect of testosterone on platelet production, and the bone marrow may exhibit greater thrombopoietic activity in males. Estrogen, on the other hand, has a more inhibitory effect in females. These variations between the sexes could be a natural physiological response or a normal biological factor [44,45,46].

Histopathological studies complement hematological and biochemical analyses. The inflammatory infiltrates observed in the liver of male and female mice treated with ethanolic extract of *Combretum micranthum* at doses of 50 mg/kg (acute toxicity) and 2000 mg/kg (subacute study) suggest a moderate inflammatory response. This reaction may be linked to immune system activation by the bioactive compounds in the extract, leading to mild hepatic inflammation, with more pronounced responses at higher doses. These changes indicate that the liver may be trying to manage mild toxicity induced by the extract, and these results may reflect an inflammatory response, suggesting a moderate immune reaction to chemical or metabolic stress caused by the extract [47]. The bacterial proliferation observed in the stomachs of mice treated with *Combretum micranthum* could indicate gastrointestinal alterations. This could be attributed to intestinal microbiota disturbances, gastric mucosa inflammation, digestive motility changes, or a weakening of local immune mechanisms, thereby promoting bacterial proliferation and gastrointestinal disorders [48,49]. The vacuoles observed in pancreatic cells may result from lipid or glycogen accumulation, mitochondrial dysfunction, or an inflammatory response to the metabolic changes induced by the plant compounds [50]. While vacuolization may be reversible and mild, it could indicate an adaptive response to the phytoconstituents, particularly at higher doses, and a temporary disruption of cellular homeostasis [51,52]. Inflammatory infiltration and renal mineralization observed in mice treated with *Combretum micranthum* may result from an inflammatory response caused by renal irritation, oxidative stress damaging the cells, or metabolic disruptions. These effects could lead to mineral deposits and functional alterations in the kidneys, suggesting renal toxicity associated with the plant extract [53].

## 4. Materials and Methods

### 4.1. Plant Material

#### 4.1.1. Harvesting and Identification

*Combretum micranthum* leaves were collected from the Sélibaby region of Mauritania. Samples, including leaves, flowers, fruits, and stems, were identified at the Herbarium of the Botany Department, University of Agronomic and Veterinary Sciences, Cluj-Napoca, Romania (Voucher No. 30431/31.10.2024). Leaves were air-dried in shade at room temperature (25 ± 3 °C) and powdered using an electric grinder.

#### 4.1.2. Preparation of the Ethanolic Leaf Extract

The extract was prepared at the Food Technology and Human Nutrition Research Laboratory, ENSA, Algiers, Algeria. A 100 g sample of powdered leaves was mixed with 2000 mL of absolute ethanol (70%) and stirred for 2 h. The extract was filtered using Whatman filter paper and centrifuged at 3000 rpm for 12 min. The filtrate was evaporated at 50 °C using a rotary evaporator, and the resultant dry extract was stored at 4 °C. The extraction yield was calculated using the following formula:

Yield (%) = [Final dry extract weight/Initial dry plant weight] × 100

#### 4.1.3. Phenolic Compound Characterization by HPLC-DAD-ESI/MS

The phenolic composition of the extract was analyzed using the HPLC-DAD-ESI-MS method described by Călinoiu, L.F, and Vodnar, [53], with slight modifications. The sample was filtered through a Chromafil Xtra nylon membrane filter (0.45 µm), and 20 μL was injected into the HPLC system (Agilent 1200, Agilent Technologies, Santa Clara, CA, USA) equipped with a quaternary pump, solvent degasser, autosampler, UV-Vis diode array detector (DAD), and a single quadrupole mass spectrometer (model 6110). Separation was performed on a Kinetex XB-C18 column (5 µm; 4.6 × 150 mm, Phenomenex, Torrance, CA, USA). The mobile phases consisted of (A) water with 0.1% acetic acid and (B) acetonitrile with 0.1% acetic acid. The gradient elution program was as follows: 0–2 min, 5% B; 2–18 min, linear increase to 40% B; 18–20 min, increase to 90% B; held at 90% B until 24 min; decreased to 5% B at 25 min and held until 30 min. The flow rate was set at 0.5 mL/min, and the column oven temperature was maintained at 25 ± 0.5 °C. Spectral data were collected across the 200–600 nm range for all peaks, and chromatograms were monitored at λ = 280 nm and 340 nm. Mass spectrometric detection was performed in positive electrospray ionization (ESI+) full scan mode under the following conditions: capillary voltage 3000 V, drying gas temperature 350 °C, nitrogen flow rate 7 L/min, and m/z scan range of 120–1200. Data was acquired and interpreted using Agilent ChemStation software, version Rev B.02.01-SR2 [260]. Quantification of phenolic compounds was performed using external calibration curves generated from authentic standards, demonstrating excellent linearity (R^2^ > 0.99).

### 4.2. Experimental Animals

BALB/c mice (male and female), aged 8–10 weeks, were sourced from the Institutul Național de Cercetare-Dezvoltare Medico-Militară “Cantacuzino” (Bucharest, Romania). Animals were housed in plastic cages under controlled conditions (23–25 °C, 55 ± 10% humidity, and a 12-h light/dark cycle) with free access to food and water. Following a 14-day acclimatization period, experiments complied with European Directive 2010/63/EU and Romanian Law 43/2014. Ethical approval was granted by the Ethics Committee of the University of Agronomic Sciences and Veterinary Medicine, Cluj-Napoca, Romania (Approval No. 429/27.02.2024). Ethical probation was also granted by the National Veterinary and Food Safety Authority (No. 402 dated 12.04.2025)

#### 4.2.1. Acute Oral Toxicity Assessment (14 Days)

**Figure 4:**
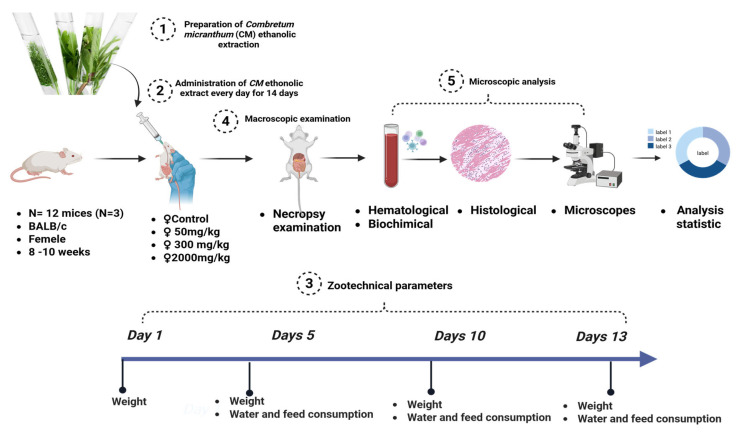
Schematic overview of the acute toxicity assessment protocol.

Twelve healthy female BALB/c mice (19.16 ± 0.14 g) were divided into four groups (n = 3 per group) following OECD Guideline 425 [54]. Three groups received doses of 50 mg/kg, 300 mg/kg, and 2000 mg/kg of *Combretum micranthum* ethanolic extract via gavage, while the control group received distilled water. Observations for behavioral and clinical changes (e.g., aggression, salivation, tremors, lethargy, unusual locomotion) were recorded during the first 30 min, every hour for 5 h, and periodically for 48 h. Weight, food/water intake, and mortality were monitored daily for 14 days. At the end of the study, animals were anesthetized with isoflurane [55], and blood samples were collected for hematological and biochemical analysis. Organs were weighed and preserved in 10% formalin for subsequent histopathological examination. A schematic representation of the acute toxicity assessment protocol is provided in Figure 4.

#### 4.2.2. Subacute Oral Toxicity Assessment (28 Days)

**Figure 5:**
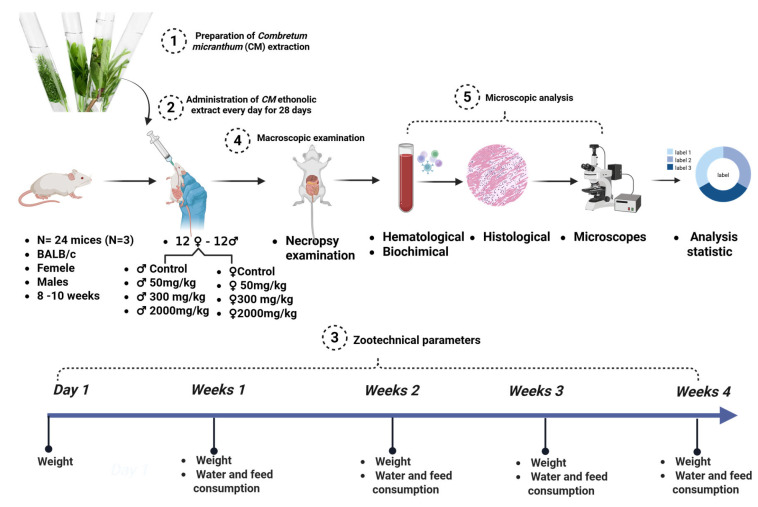
Schematic overview of the subacute toxicity assessment protocol.

Twenty-four BALB/c mice (3 males and 3 females per group) were divided into four groups following OECD Guideline 407 [56], modified by [20,57]. Doses of 50 mg/kg, 300 mg/kg, and 2000 mg/kg of *Combretum micranthum* ethanolic extract were administered daily via gavage for 28 days, while the control group received distilled water. Observations for behavioral and physiological changes were recorded daily. On day 29, animals were anesthetized, and blood was collected. Organs were weighed and fixed in 10% formalin for subsequent histopathological analysis. A summary of the subchronic toxicity protocol is depicted in Figure 5.

### 4.3. Anesthesia and Euthanasia Procedures

Animals were deeply sedated using an overdose of isoflurane, administered via a sealed induction chamber via inhalation, to ensure uniform exposure. Once a lack of reflex responses confirmed deep anesthesia, cervical dislocation was performed as a secondary method to confirm euthanasia. This procedure was conducted in strict accordance with Directive 2010/63/EU of the European Parliament and of the Council of 22 September 2010 on the protection of animals used for scientific purposes, ensuring compliance with established ethical standards [58,59].

### 4.4. Blood and Organ Sampling

Blood samples were collected in ethylenediaminetetraacetic acid (EDTA) tubes for hematological analysis and heparinized tubes for biochemical assays. Organs (liver, kidney, spleen, lungs, stomach, pancreas, intestines, and heart) were preserved in 10% formalin for histological analysis.

### 4.5. Hematological and Biochemical Analyses

Hematological parameters were analyzed using an automatic hematological analyzer (Diatron Abacus Junior 5, Budapest, Hungary). The measured parameters were as follows: white blood cells (WBC), lymphocytes (LYM), monocytes (MON), neutrophils (NEU), lymphocyte percentage (LY), monocytes percentage (MON), neutrophils percentage (NE), red blood cells (RBC), hemoglobin (HGB), hematocrit (HCT), mean corpuscular volume (MCV), mean corpuscular hemoglobin (MCH), mean corpuscular hemoglobin concentration (MCHC), red blood cell distribution width (RDWC), platelets (PLT), plateletcrit (PCT), and mean platelet volume (MPV).

The biochemical analysis was performed using an automatic chemical analyzer (Scil—Element RC, Viernheim, Germany). The measured parameters were as follows: albumin (ALB), total proteins (TP), globulins (GLOB), albumin/globulin ratio (A/G), total bilirubin (TB), alanine transaminase (ALT), alkaline phosphatase (ALP), amylase (AMY), creatinine (CREA), urea (UREA), glucose (GLU), serum calcium (Ca), phosphate (PHOS), potassium (K^+^) and sodium (Na^+^).

### 4.6. Histopathological Analysis

The harvested tissues were fixed in 10% formalin for 48 h. After fixation, the samples were dehydrated and clarified, which was achieved by immersion in ethyl alcohol baths with increasing concentration, respectively, in xylene baths. After clarifying the samples, the samples’ infiltration with paraffin was carried out at 58 °C for 5 h, using a paraffin with a low melting temperature. After this, thin sections of 2 µm were obtained from the paraffin blocks using the rotary microtome. According to a routine protocol, they were later stained using hematoxylin–eosin (H&E). Histological samples were examined under an Olympus BX51 microscope (Olympus Life and Material Science Europa GMBH, Hamburg, Germany), and the bright field images were obtained with an Olympus SP350 (Olympus Life and Material Science Europa GMBH) digital camera and processed using the Olympus Cell Sens software (version 2.1). The Group Severity Degree Score is calculated by dividing the total severity scores for a lesion within a group by the number of tests examined.

### 4.7. Statistical Analysis

Data are presented as mean ± standard deviation (SD). Statistical analysis was performed using GraphPad Prism 10 (San Diego, CA, USA). One-way ANOVA followed by Tukey’s post hoc test was used for parametric comparisons, with a *p*-value < 0.05 regarded as statistically significant.

## 5. Conclusions

In this study, oral administration of the ethanolic extract of *Combretum micranthum* leaves at doses up to 2000 mg/kg did not cause any mortality, either during the acute toxicity evaluation (14 days) or the subacute toxicity evaluation (28 days) in BALB/c mice. Furthermore, no clinical changes or significant variations in body weight were observed. Significant changes (*p* < 0.001) were noted in some hematological and biochemical parameters in the treated groups compared to the control groups. Histopathological examination revealed minimal alterations in certain organs (livers, stomach, and pancreas) of the treated animals (represented by inflammatory cell infiltrate within the liver, gastric bacterial overgrowth, and renal medullary mineralization). These results do not confirm that oral administration of the ethanolic extract of *Combretum micranthum* leaves at doses up to 2000 mg/kg induces toxicity. Instead, they open new perspectives for future research, suggesting that one should not rely solely on clinical signs, macroscopic appearance, or the number of deceased animals to determine the toxic dose. It is essential to analyze further other crucial biological parameters, including biochemical, hematological, and histopathological parameters. Thus, this study suggests the need for more in-depth investigations to better understand the biochemical, immunological, and hematological responses, particularly regarding the administration of higher doses of *Combretum micranthum* extract, and to explore in detail their underlying mechanisms and physiological implications.

## Figures and Tables

**Table 1:** Yield of Ethanolic Extract of *Combretum micranthum*.

Plant Material	Initial Mass of Plant (g)	Mass of Dry Extract (g)	Yield (%)
*Combretum micranthum*	100	17.34	17.34

**Table 2:** The content of phenolic compounds in ethanolic extracts of *C.micranthum* leaves by HPLC-DAD-ESI-MS (mg/g of extract).

	Phenolic Compounds	Subclass	R_t_ (min)	λmax (nm)	[M+H]^+^ (*m*/*z*)	Extract (mg/g)
1	Gallic acid	Hydroxybenzoic acid	4.70	275	171	4.80
2	Protocatechuic acid	Hydroxybenzoic acid	9.01	280	155	14.14
3	1,6-Digalloyl-glucose	Gallotannin	13.16	280	485	5.82
4	Ellagic acid-arabinoside	Hydroxybenzoic acid	13.93	270, 360	435	14.04
5	Ellagic acid-glucoside	Hydroxybenzoic acid	14.43	270, 360	465	8.08
6	Sanguiin H-4	Ellagitannin	15.14	270, 360	635	102.56
7	Corilagin	Ellagitannin	16.22	270, 360	635	63.29
8	Ellagic acid	Hydroxybenzoic acid	16.63	270, 360	303	12.10
9	Combretastatin B1	Stilbene	22.69	275	335	68.71
	Total phenolics (mg/g)					293.54

**Table 3:** Organ weights (g) of BALB/c female mice in the acute oral toxicity study (mean ± SD).

Organs(g)	Control	50 mg/kg	300 mg/kg	2000 mg/kg
Lung	0.25 ± 0.03	0.25 ± 0.03	0.25 ± 0.02	0.25 ± 0.01
Spleen	0.09 ± 0.01	0.09 ± 0,01	0.09 ± 0.0	0.09 ± 0
Kidney	0.25 ± 0.04	0.25 ± 0.02	0.25 ± 0.02	0.25 ± 0.04
Liver	0.96 ± 0	0.96 ± 0.07	0.96 ± 0.01	0.96 ± 0.05
Heart	0.13 ± 0.03	0.13 ± 0.06	0.13 ± 0.04	0.13 ± 0

**Table 4:** Biochemical parameters of BALB/c female mice following acute oral administration of *Combretum micranthum* extract.

Parameters	Control	50 mg/kg	300 mg/kg	2000 mg/kg
ALB (g/dL)	3.15 ± 0.07	3.10 ± 0.15	3.16 ± 0.05	3.10 ± 0.14
TP (g/dL)	5.20 ± 0.14	5.25 ± 0.07	5.20 ± 0.14	5.20 ± 0.28
GLOB (g/dL)	2.20 ± 0.10	2.20 ± 0.07	2.25 ± 0.07	2.25 ± 0.07
A/G	1.52 ± 0.01	1.55 ± 0.01	1.51 ± 0.02	1.51 ± 0.02
TB (g/dL)	0.25 ± 0.07	0.25 ± 0.07	0.23 ± 0.05	0.20 ± 0
ALT (u/L)	112.33 ± 0.57	111.33 ± 0.57	111.66 ± 0.57	112.96 ± 1.15
ALP (u/L)	140.66 ± 0.57	141 ± 0.13	141.66 ± 0.57	141.66 ± 0.57
AMY (u/L)	2714.66 ± 11.15	2734.66 ± 10.57 ***	2731.66 ± 12.57 ***	2739.66 ± 18.57 ***
CREA (mg/dL)	0.10 ± 1.69	0.10 ± 0.69	0.10 ± 0.09	0.1± 0.04
UREA (mg/dL)	43.74 ± 0	43.45 ± 0.02	43.25 ± 0.07	43.58 ± 0.03
GLU (mg/dL)	132.61 ± 0.01	132.81 ± 0.01	132.39 ± 0.01	132.80 ± 0
Ca (mg/dL)	9.14 ± 0.02	9.16 ± 0	9.11 ± 0	9.13 ± 0
PHOS (mg/dL)	5.85 ± 0	5.88 ± 0	5.83 ± 0	5.86 ± 0
K^+^ (mmol/L)	6.31 ± 0.01	6.36 ± 0.02	6.36 ± 0.01	6.34 ± 0.01
Na^+^ (mmol/L)	148.43 ± 0.15	148.40 ± 0.14	148.46 ± 0.40	148.45 ± 0.07

Data are expressed as mean ± standard deviation. Significant differences compared to the control group are indicated as follows: *** *p* < 0.001.

**Table 5:** Hematological parameters of BALB/c female mice after acute oral administration of *Combretum micranthum* extract.

Parameters	Control	50 mg/kg	300 mg/kg	2000 mg/kg
WBC (10^9^/L)	3.33 ± 0	3.31 ± 0	3.33 ± 0	3.34 ± 0.02
LYM (10^9^/L)	2.54 ± 0	2.5 ± 0.02	2.53 ± 0.01	2.51 ± 0.01
MON (10^9^/L)	0.11 ± 0	0.12 ± 0.02	0.12 ± 0.01	0.11 ± 0.02
NEU (10^9^/L)	0.57 ± 0.01	0.57 ± 0	0.56 ± 0.01	0.56 ± 0.02
LY (%)	78.45 ± 0.07	78.41 ± 0.14	78.41 ± 0.14	78.4 ± 0.14
MO (%)	4.32 ± 0.16	4.29 ± 0.14	4.30 ± 0.28	4.30± 0.14
NE (%)	16.20 ± 0.14	16.15 ± 0.07	16.25 ± 0.07	16.25 ± 0.21
RBC (10^12^/L)	9.44 ± 0.02	9.43 ± 0.02	9.43 ± 0.04	9.44 ± 0.02
HGB (g/dL)	14.35 ± 0.21	14.4 ± 0.14	14.35 ± 0.21	14.33 ± 0.20
HCT (%)	52.27 ± 0.02	52.4 ± 0.02	52.70 ± 0	54.21 ± 0.01
MCV	54.66 ± 0.57	54.56 ± 0.57	54.33 ± 2.08	54.63 ± 0.57
MCH (pg.)	15.25 ± 0.21	15.25 ± 0.21	15.20 ± 0.14	15.25 ± 0.21
MCHC (g/dL)	26.55 ± 0.07	26.65 ± 0.07	26.43 ± 0.14	26.51 ± 0.14
RDWC (%)	17.14 ± 0.14	17.15 ± 0.21	17.15 ± 0.21	17.17 ± 0.14
PLT (10^9^/L)	574 ± 3.60	372 ± 4.35 ***	335.66 ± 2.51 ***	380.66 ± 1.52 ***
PCT (%)	0.44 ± 0.01	0.44 ± 0.01	0.44 ± 0.01	0.43 ± 0.02
MPV (%)	8.10 ± 0.14	8.15 ± 0.07	8.20 ± 0.14	8.12 ± 0.21
PDWC (%)	35.05 ± 0.07	35.15 ± 0.07	35.05 ± 0.07	35.40 ± 0.14

Data are expressed as mean ± standard deviation. Significant differences compared to the control group are indicated as follows: *** *p* < 0.001.

**Table 6:** Histopathological profile of BALB/c female mice in the acute toxicity study. Incidence (marked by the round brackets) and severity scores (marked by the square brackets) of observed lesions are provided (as a group average) for each organ/group.

Organs	Histological Changes	Groups			
		Control	50 mg/kg	300 mg/kg	2000 mg/kg
Liver	Inflammatory infiltrate cells (lymphocytes and neutrophils)	(0)	(2) [0.66]	(0)	(3) [1]
Pancreas	Vacuolization	(0)	(0)	(1) [0.33]	(0)
Stomach	Bacterial Overgrowth	(0)	(1) [0.33]	(2) [0.66]	(0)
Kidneys	Medullary mineralization	(0)	(3) [1]	(0)	(0)
	Inflammatory infiltrate cells (lymphocytes)	(0)	(0)	(1) [1]	(0)

**Table 7:** Organ weights (g) of BALB/c male mice in the subacute toxicity study (mean ± SD).

Organs(g)	Control	50 mg/kg	300mg/kg	2000 mg/kg
Lung	0.16 ± 0	0.16 ± 0.03	0.16 ± 0.05	0.16 ± 0.08
Spleen	0.38 ± 0.42	0.38 ± 0.05	0.38 ± 0.01	0.38 ± 0.04
Kidney	0.42 ± 0.06	0.42 ± 0.02	0.42 ± 0.03	0.42 ± 0.29
Liver	0.80 ± 0.94	0.80 ± 0.16	0.80 ± 0,17	0.80 ± 0.73
Heart	0.12 ± 0.02	0.12 ± 0.02	0.12 ± 0.02	0.12 ± 0.09

**Table 8:** Organ weights (g) of BALB/c female mice in the subacute toxicity study (mean ± SD).

Organs(g)	Control	50 mg/kg	300 mg/kg	2000 mg/kg
Lung	0.17 ± 0.05	0.17 ± 0,06	0.17 ± 0.01	0.17 ± 0.07
Spleen	0.09 ± 0	0.94 ± 0,01	0.93 ± 0.01	0.94 ± 0
Kidney	0.20 ± 0.04	0.20 ± 0,01	0.20 ± 0.02	0.20 ± 0
Liver	0.98 ± 0.20	0.98 ± 0,04	0.98 ± 0.09	0.98 ± 0
Heart	0.09 ± 0	0.096± 0.01	0.09 ± 0.02	0.09 ± 0

**Table 9:** Hematology profile of females BALB/c mice in subacute toxicity evaluation.

Parameters	Control	50 mg/kg	300 mg/kg	2000 mg/kg
WBC (10^9^/L)	1.54 ± 0.001	1.55 ± 0.7	1.54 ± 0.2	1.53 ± 0.08
LYM (10^9^/L)	1.16 ± 0.03	1.17 ± 0.20	1.17 ± 0	1.16 ± 0.02
MON (10^9^/L)	0.06 ± 0	0.05 ± 0	0.06 ± 0	0.06 ± 0
NEU (10^9^/L)	0.30 ± 0	0.32 ± 0.43	0.31 ± 0	0.31 ± 0
LY (%)	76.0 ± 0.20	50 ± 0.90 ***	65.8 ± 0.64 ***	69.7 ± 0.90 **
MO (%)	4.20 ± 0.40	4.20 ± 0.20	4.30 ± 0.23	4.30 ± 0.50
NE (%)	19.50 ± 0.90	19.50 ± 0.45	19.50 ± 0.27	19.30 ± 0.26
RBC (10^12^/L)	9.38 ± 0.40	9.37 ± 0.20	9.39 ± 0.60	7.36 ± 0.65
HGB (g/dL)	14.30 ± 0.25	14.60 ± 0.65	14.50 ± 0.30	14.70 ± 0.32
HCT (%)	51.08 ± 0.86	36.80 ± 0.25 ***	27.84 ± 0.23 ***	30.65 ± 0.63 ***
MCV	54 ± 0.32	53 ± 0.49	54 ± 0.15	54 ± 0.28
MCH (pg)	15.30 ± 0.84	15.40 ± 0.34	15.40 ± 0.18	15.50 ± 0.23
MCHC (g/dL)	28.10 ± 0.16	28.60 ± 0.26	28.2 ± 0.34	28.80 ± 0.91
RDWC (%)	17.20 ± 0.27	17.03 ± 0.27	17.3 ± 0.62	17.50 ± 0.11
PLT (10^9^/L)	625 ± 3.83	533 ± 3.60 ***	286 ± 4.20 ***	220 ± 2.10 ***
PCT (%)	0.50 ± 0.03	0.50 ± 0.01	0.51 ± 0.08	0.50 ± 0
MPV (%)	8.0 ± 0.73	8.10 ± 0.56	8.10 ± 0.49	8.10 ± 0.13
PDWC (%)	32.60 ± 0.20	32.70 ± 0.20	32.80 ± 0.34	32.20 ± 0.28

Data are expressed as mean ± standard deviation. Significant differences compared to the control group are indicated as follows: ** *p* < 0.01, *** *p* < 0.001.

**Table 10:** Hematology profile of male BALB/c mice in subacute toxicity evaluation.

Parameters	Control	50 mg/kg	300 mg/kg	2000 mg/kg
WBC (10^9^/L)	1.69 ± 0	1.67 ± 0.5	1.66 ± 0	1.65 ± 0.02
LYM (10^9^/L)	1.05 ± 0	1.05 ± 0.43	1.06 ± 0.02	1.05 ± 0.01
MON (10^9^/L)	0.16 ± 0	0.16 ± 0	0.15 ± 0.06	0.15 ± 0
NEU (10^9^/L)	0.48 ± 0.02	1.48 ± 0	0.47 ± 0.03	0.48 ± 0.02
LY (%)	62.2 ± 05	39.2 ± 0.23 ***	24.3 ± 0.3 ***	37.2 ± 0.60 ***
MO (%)	9.6 ± 0.20	9.6 ± 0.45	9.4 ± 0.7	9.50 ± 0,60
NE (%)	28.2 ± 0.16	28.30 ± 0.12	28.3 ± 1.20	28,4 ± 0,50
RBC (10^12^/L)	9.64 ± 0.01	9.62 ± 0.66	9.62 ± 0.40	9.65 ± 0.40
HGB (g/dL)	14.1 ± 0.20	14.10 ± 0.33	14.20 ± 0.20	14.20 ± 0,10
HCT (%)	51.51 ± 0.7	59.89 ± 0.51 ***	60.38 ± 0.14 ***	59.88 ± 0.25 ***
MCV	53 ± 0.30	53 ± 0.45	53 ± 0.43	54 ± 0.67
MCH (Pg)	14.6 ± 0.4	14.50 ± 0.40	14.50 ± 0.34	14.70 ± 0.43
MCHC (g/dL)	27.3 ± 0.67	27.0 ± 0.10	27.50 ± 0.34	27.0 ± 0.29
RDWC (%)	18.0 ± 0.34	18.0 ± 0.45	18.50 ± 0.45	18.2 ± 0.20
PLT (10^9^/L)	347 ± 4.30	584 ± 6,10 ***	505 ± 2.14 ***	431 ± 4.20 ***
PCT (%)	0.29 ± 0.30	0.30 ± 0.03	0.29 ± 0.15	0.29 ± 0.12
MPV (%)	8.2 ± 0.01	8.30 ± 0.44	8.2 0 ± 0.45	8.30 ± 0.420
PDWC (%)	39 ± 0.90	39.80 ± 0.65	39.80 ± 0.65	39.40 ± 0.52

Data are expressed as mean ± standard deviation. Significant differences compared to the control group are indicated as follows: *** *p* < 0.001.

**Table 11:** Biochemical parameters of BALB/c female mice following subacute oral administration of *Combretum micranthum* extract.

Parameters	Control	50 mg/kg	300 mg/kg	2000 mg/kg
ALB (g/dL)	3 ± 0.03	3.10 ± 0.90	3 ± 0.3	3.10 ± 0.10
TP (g/dL)	5.0 ± 0.15	5.15 ± 0.90	5.1 ± 0.13	5.10 ± 0.19
GLOB (g/dL)	2.20 ± 0.90	2.30 ± 0.06	2.2 ± 0.9	2.20 ± 0.04
A/G	1.56 ± 0.04	1.57 ± 0.03	1.58 ± 0.04	1.57 ± 0.03
TB (g/dL)	0 ± 0	0 ± 0	0.10 ± 0	0 ± 0
ALT (u/L)	33.33 ± 0.40	93.33 ± 0.18 ***	73.0 ± 0.66***	92 ± 1.15 ***
ALP (u/L)	87.0 ± 0.67	104 ± 0.61 ***	104.66 ± 0.89 ***	103.66 ± 0.57 ***
AMY (u/L)	2156 ±13.25	2350 ± 13.37 ***	2355 ± 11.57 ***	2336 ± 90 ***
CREA (mg/dL)	0.20 ± 0.69	0.20 ± 0.76	0.20 ± 0	0.20± 0
UREA (mg/dL)	56.27 ± 0.56	44.85 ± 0.03 ***	44.10 ± 0.60 ***	29.21 ± 0.02 ***
GLU (mg/dL)	120.82 ± 0.03	240.25 ± 0.01 ***	249.45 ± 0.05 ***	240.40 ± 0.05 ***
Ca (mg/dL)	9.14 ± 0.02	9.13 ± 0	9.14 ± 0.08	9.13 ± 0.08
PHOS (mg/dL)	8.46 ± 0.05	8.46 ± 0.08	8.45 ± 0	8.45 ± 0.02
K^+^ (mmol/L)	5.25 ± 0.04	5.20 ± 0.06	5.30 ± 0.01	5.25 ± 0.01
Na^+^ (mmol/L)	149.80 ± 0.19	150 ± 0.18	150.06 ± 0.14	150.45 ± 0.07

Data are expressed as mean ± standard deviation. Significant differences compared to the control group are indicated as follows: *** *p* < 0.001.

**Table 12:** Biochemical parameters of BALB/c male mice following subacute oral administration of *Combretum micranthum* extract.

Parameters	Control	50 mg/kg	300 mg/kg	2000 mg/kg
ALB (g/dL)	3.10 ± 0.30	3.1 ± 0.21	3.10 ± 0.16	3 ± 0.14
TP (g/dL)	5.20 ± 0.20	5.3 ± 0.03	5.30 ± 0.11	5.2 ± 0.17
GLOB (g/dL)	2.70 ± 0.10	2.70 ± 0.04	2.80 ± 0.08	2.7 ± 0.09
A/G	1.11 ± 0.02	1.10 ± 0.02	1.11 ± 0.02	1.12 ± 0.06
TB (g/dL)	0 ± 0	0 ±0	0.1 ± 0	0 ± 0
ALT (u/L)	38.00 ± 0.36	107 ± 03.6 ***	181 ± 0.57 ***	142 ± 0.66 ***
ALP (u/L)	88 ± 0.12	108 ± 0.02 ***	100 ± 0.66 ***	109 ± 0.66 ***
AMY (u/L)	2196 ± 10.11	2461.17 ±09.31 ***	2453 ± 11.32 ***	2422 ± 12.57 ***
CREA (mg/dL)	0.10 ± 0.10	0.20 ± 0	0.10 ± 0	0.20 ± 0
UREA (mg/dL)	57.71 ± 0.60	50.58 ± 0.49 **	50.20 ± 0.07 **	50.18 ± 0.90 **
GLU (mg/dL)	126.03 ± 0.02	147.25 ± 0.3 ***	145.94 ± 0.04 ***	143.48 ± 0.70 ***
Ca (mg/dL)	8.99 ± 0.01	8.99 ± 0.07	8.99 ± 0.03	8.99 ± 0.04
PHOS (mg/dL)	3.77 ± 0.07	3.76 ± 0.05	3.74 ± 0.02	3.77 ± 0.02
K^+^ (mmol/L)	6.49 ± 0.04	6.51 ± 0.03	6.49 ± 0.03	6.51 ± 0.05
Na^+^ (mmol/L)	150.50 ± 0.10	149.20 ± 0.11	151 ± 0.24	149.90 ± 0.08

Data are expressed as mean ± standard deviation. Significant differences compared to the control group are indicated as follows: ** *p* < 0.01, *** *p* < 0.001.

**Table 13:** Histopathological profile of BALB/c female and male mice in the subacute toxicity study. Incidence (marked by the round brackets) and severity scores (marked by the square brackets) of observed lesions are provided (as a group average) for each organ/group.

Organs	Histological Changes	Groups							
		Control		50 mg/kg		300 mg/kg		2000 mg/kg	
		♂	♀	♀	♂	♀	♂	♀	♂
Liver	Inflammatory infiltrate cells (lymphocytes and neutrophils)	(0)	(0)	(0)	(1) [0.33]	(0)	(0)	(1) [0.33]	(2) [0.33]
Pancreas	Vacuolization	(0)	(0)	(0)	(0)	(0)	(2) [0.33]	(0)	(0)
Stomach	Bacterial Overgrowth	(0)	(0)	(2) [0.33]	(2) [0.66]	(0)	(0)	(2) [0.33]	(0)

## Data Availability

The data are contained in the article.
